# Acute Exposure to Traces of Chlorantraniliprole Does Not Alter Antipredator Behaviour in Wild Crickets

**DOI:** 10.1002/ece3.74064

**Published:** 2026-07-21

**Authors:** Emily Gilford, Miranda Johnstone, Mark Pitt, Rolando Rodríguez‐Muñoz, Chris Bass, Tom Tregenza, Bram Kuijper

**Affiliations:** ^1^ Centre for Ecology and Conservation University of Exeter Penryn UK; ^2^ School of Biodiversity, One Health and Veterinary Medicine University of Glasgow Glasgow UK

**Keywords:** anthropogenic stressors, anti‐predator behaviour, insect behaviour, pesticide exposure, sublethal effects, wild populations

## Abstract

Exposure to sublethal concentrations and low, non‐target pesticide residues is now a common scenario for insects sharing their habitat with human agriculture. Such exposures have the potential to disrupt behaviour. However, much of this evidence comes from laboratory studies under controlled conditions and is concentrated on a limited range of taxa and pesticide classes, particularly pollinators. We tested whether exposure to a low, residue‐level concentration of chlorantraniliprole, a widely used insecticide known for its long environmental persistence, affected antipredator escape behaviours in wild field crickets (*Gryllus campestris*). Adult crickets were treated with either pesticide or a sham control. They then underwent behavioural tests in the wild. Following a simulated predator attack, we measured two key behaviours: escape speed and subsequent latency to re‐emerge from their burrow. We found no consistent effects of pesticide exposure on either behaviour. Re‐emergence latency increased over successive days across both groups, while escape speed did not change. Our findings indicate that a single, acute, residue‐level exposure to chlorantraniliprole did not measurably alter the anti‐predator behaviours we studied during the days immediately after exposure. Our study contributes one piece of the broader jigsaw needed to understand the behavioural effects of environmentally relevant pesticide exposure in nature.

## Graphical abstract

We tested whether acute, residue‐level exposure to a widely used insecticide alters antipredator behaviour in wild field crickets. Using a field‐based behavioural assay, we found no detectable effects on escape speed or re‐emergence latency over the days following exposure. These results suggest that under this exposure scenario, pesticide effects on behaviour may be weaker or more context‐dependent than often assumed.

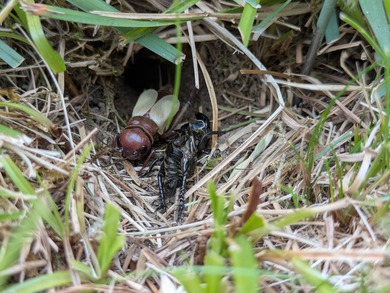

## 1 Introduction

Natural systems are increasingly shaped by anthropogenic disturbance, exposing wild animals to a range of novel and recurrent challenges. Insects, which underpin terrestrial ecosystems, are experiencing alarming declines, with over 40% of species threatened with extinction globally due to agricultural intensification, agrochemical pollution, and climate change (Sánchez‐Bayo and Wyckhuys 2019). Pesticides are now routinely detected on non‐target insects (i.e., species not intended to be controlled by pesticide applications). This is the case even in semi‐natural landscapes and conserved habitats far from areas of direct application, through processes such as spray drift, runoff, and atmospheric deposition (Albaseer et al. 2025; Brühl et al. 2021; Quandahor et al. 2024; Wan et al. 2025). These pervasive residues highlight that pesticide exposure is widespread, persistent, and rarely limited to target pest species.

Non‐target insects perform essential ecosystem services, including pollination, natural pest control, and contributions to nutrient cycling; all of which can be disrupted by pesticide contamination (Albaseer et al. 2025; Goulson 2013). Field surveys have documented the presence of pesticide mixtures on pollinators and predatory insects in agricultural margins and protected areas, suggesting that biodiversity losses are not explained solely by habitat conversion, but also by chemical drift and chronic contamination (Brühl et al. 2021; Sánchez‐Bayo and Wyckhuys 2019). Consequently, concern is growing not only about the lethal effects of pesticide use, but also about potential sublethal consequences for behaviour, physiology, and reproduction in wild insect populations.

While acute mortality remains the primary measure in pesticide risk assessment, there is increasing recognition that survival alone does not capture the ecological impact of exposure, particularly when measured in the laboratory. This has prompted growing interest in alternative measures of sublethal effects of pesticides which may reveal impairment in insects long before death. In ecotoxicology, “sublethal” exposure generally refers to pesticide concentrations that do not cause substantial acute mortality but can still induce physiological or behavioural impairments (Bertram et al. 2022; Desneux et al. 2007; Gandara et al. 2024). Sublethal pesticide exposure can alter physiology or behaviour by impairing movement, reproduction, or sensory perception, ultimately influencing fitness and population persistence (Gandara et al. 2024; Guedes et al. 2025) and has been proposed as a major driver of global insect biodiversity loss (van der Sluijs 2020).

Despite the prevalence of pesticide drift and contamination, the majority of research on sublethal pesticide effects has been conducted in controlled laboratory environments, often on a select suite of model taxa, including a particularly strong focus on bees and bumblebees, and under simplified conditions. Field and semi‐field based studies in pollinators have demonstrated that sublethal pesticide exposure can impair ecologically relevant behaviours such as foraging efficiency, navigation and pollination services (Rundlöf et al. 2015; Stanley, Garratt, et al. 2015; Woodcock et al. 2017). Such work provides critical mechanistic insight, but still represents a relatively limited subset of ecological contexts compared to the diversity of insect taxa and environments in which pesticide exposure occurs. In these systems, multiple interacting stressors may modify both pesticide toxicity and behavioural responses (Brühl et al. 2021; Kaunisto et al. 2016; Slos and Stoks 2008; Wan et al. 2025), yet such effects are poorly understood.

Understanding how sublethal pesticide exposure affects the behaviour of wild insects is particularly urgent, as declines are increasingly reported in landscapes with little or no direct pesticide application (Albaseer et al. 2025; Brühl et al. 2021; Sánchez‐Bayo and Wyckhuys 2019). In natural environments, pesticide effects may interact strongly with environmental variables such as temperature and humidity, with elevated temperatures often amplifying toxicity and sublethal effects, and lower humidity enhancing insect susceptibility to pesticides by increasing metabolic and desiccation stress (Tsaganou et al. 2021; Verheyen and Stoks 2019). Other work has highlighted interactions with stressors such as nutritional stress, which may amplify pesticide impacts on survival and physiology (Tosi et al. 2017). Sublethal effects may also vary over time as individuals recover from, compensate for, or accumulate exposure impacts (Batool et al. 2024; Siviter et al. 2018). This highlights the importance of assessing behavioural responses across multiple time points following exposure, and within environmentally realistic conditions that capture natural variation in temperature, humidity, and resource availability.

Studies conducted under more ecologically realistic conditions are therefore needed to test how low‐level exposures alter ecologically relevant behaviours. Although field and semi‐field studies of pesticide effects on behaviour are well established in pollinators, particularly bees and bumblebees, these studies are largely concentrated within a limited number of taxa and chemical classes, and comparable work in other insect groups remains scarce. Existing work focusses on a relatively narrow set of chemical groups, especially pyrethroids, organophosphates and neonicotinoids, with fewer studies examining other taxa or newer insecticide classes under natural conditions (Biondi et al. 2013; Desneux et al. 2007; Guedes et al. 2025; Siviter et al. 2021). Expanding this evidence base is essential for linking laboratory‐derived mechanisms to behavioural and demographic outcomes in more natural systems.

Here we develop a field assay to test how a single, acute, ecologically relevant pesticide exposure influences antipredator behaviour in a wild insect population. In the context of natural populations, movement related traits, such as flight, walking, or burrowing, are particularly informative for understanding sublethal pesticide effects as they link directly to ecological performance (Crall et al. 2018; Desneux et al. 2007). Mobility determines how insects locate food and find mates, and even subtle changes can have cascading effects on survival and fitness. Moreover, movement lies at the heart of antipredator strategies, as the ability to detect, evaluate and respond to threats is highly consequential to survival. Changes in mobility may therefore compromise risk‐avoidance capacity and alter trade‐offs between predator avoidance and other activities such as foraging and reproduction. Escape behaviours are valuable indicators, as they integrate sensory, motor and motivational processes. Impaired escape may make individuals either overly responsive and wasteful of energy, or less responsive and vulnerable, depending on how pesticides disrupt neuromuscular and sensory processing (S. A. Adamo 2012; Gandara et al. 2024; Guedes et al. 2025). Laboratory experiments and recent syntheses show that sublethal insecticide exposure can impair escape responses across a range of aquatic and terrestrial organisms (Ågerstrand et al. 2020; Bertram et al. 2022; Kavallieratos et al. 2024). This suggests that sublethal pesticide effects on fitness may be mediated through alterations in escape behaviours in response to threats and stressors. However, studies under natural conditions are essential to properly gauge the effects of pesticides on escape behaviours.

To address this gap, we investigated whether acute sublethal exposure to chlorantraniliprole alters antipredator escape behaviour in wild field crickets (*Gryllus campestris*). *G. campestris* is a well‐studied orthopteran whose ecology and individual life histories have been monitored in long‐term field populations (Rodríguez‐Muñoz et al. 2010, 2019, 2025), making it an ideal model for examining pesticide effects in natural environments. Crickets exhibit a range of antipredator responses that are easily quantified in the field, including fleeing into burrows and the timing of subsequent re‐emergence. We focused on two behaviours with distinct ecological roles: escape speed, representing locomotor performance during fleeing, and re‐emergence latency, reflecting post‐disturbance risk assessment. Both behaviours were elicited using a simulated predator attack; a vibration caused by dropping a ball down a tube which landed close to the burrow entrance, which reliably triggers a flee response in this species (Gilford et al. 2026; Li et al. 2025, 2026).

We tested whether acute, sublethal chlorantraniliprole exposure influenced these escape behaviours and whether any effects persisted across two consecutive days of testing. Diamides such as chlorantraniliprole represent a rapidly expanding, multi‐billion dollar class of insecticides that are increasingly replacing neonicotinoids in agriculture (Jeanguenat 2013). Acting through ryanodine receptor modulation, diamides disrupt muscle function in target pests, while showing relatively low toxicity to vertebrates and other beneficial insects (Cordova et al. 2006; Nauen 2006). Yet, despite the widespread adoption of diamides, little is known about their sublethal or behavioural effects on non‐target species. Chlorantraniliprole, one of the most widely used diamides in Europe, dominates insecticide applications in UK orchards (AFBI 2022; Jeanguenat 2013; Ridley et al. 2020) and is marketed as a reduced‐risk, “bee‐safe” alternative to neonicotinoids (Larson et al. 2013). However, emerging research supports subtle locomotor and reproductive impairments in bees and moths following exposure (Henry et al. 2012; Zhang et al. 2022). For example, sublethal exposure to chlorantraniliprole has been shown to induce long‐lasting locomotor impairments in honey bees, including reduced movement and delayed recovery following exposure (Kadala et al. 2019), consistent with its effects on calcium signalling pathways in muscle and neural tissue. Chlorantraniliprole is also used extensively to control orthopteran pests such as rangeland grasshoppers and Mormon crickets ( *Anabrus simplex* ) (USDA APHIS 2008), yet its sublethal effects on these groups, and other non‐target orthopterans, remain poorly understood.

We focus on a single acute exposure scenario. By integrating ecologically relevant behavioural measures with a free‐living field population, our study provides one of the first assessments of how sublethal pesticide exposure shapes antipredator responses in a wild insect. Based on laboratory findings that chlorantraniliprole disrupts muscle function in invertebrates (Cordova et al. 2006; Kadala et al. 2019; Nauen 2006), we predicted that sublethal exposure would impair antipredator escape behaviour in crickets by reducing locomotor performance and altering risk assessment. Specifically, we expected dosed crickets to exhibit slower escape speeds and longer re‐emergence latencies compared to sham dosed controls. If sublethal effects accumulate or persist, we further predicted stronger behavioural impairments on the second day of testing. Conversely, if crickets rapidly recover following acute exposure, treatment effects would be transient or absent.

## 2 Methods

### 2.1 Dose Selection

A preparatory laboratory bioassay was conducted to identify a suitable chlorantraniliprole exposure concentration for use in the field experiment. As dose selection had to be carried out in the laboratory, we used the closely related species *Gryllus bimaculatus* (see Appendix 1). In the absence of species‐specific data, ecological study design often relies on information from related species or closely related study systems to inform experimental design, for example when deriving expected effect sizes or variance estimates for power analyses (Nakagawa et al. 2024). We therefore treated the use of *G. bimaculatus* as a practical working hypothesis for dose calibration rather than evidence of equivalent pesticide sensitivity between species. For the purposes of this study, we considered an operationally sublethal exposure to be one that produced modest acute mortality while remaining substantially below estimated direct‐spray exposure levels. Such exposure levels are commonly used in ecotoxicology to investigate behavioural and physiological effects while avoiding substantial acute mortality (Bertram et al. 2022; Brühl et al. 2021; Desneux et al. 2007). Full laboratory bioassay methods, dose‐selection calculations and results are provided in Appendix 1.

### 2.2 Natural Study System

Our study site is a meadow in northern Spain, in which a population of the field cricket *G. campestris* has been observed by the “Wild Crickets” project since 2006 (see www.wildcrickets.org) (Tregenza et al. 2022). *G. campestris* is flightless, and spends most of their lives around self‐dug burrows, which serve as refuges from predators throughout development and during winter diapause (Vrenozi and Uchman 2020). Crickets typically forage and sun‐bask near burrow entrances, retreating underground in response to predators, thermal extremes, or rain (Gardner et al. 2024). Nymphs emerge in spring, become more active as they develop, and reach adulthood in April–May (Rodríguez‐Muñoz et al. 2011). The species is sensitive to substrate‐borne vibration (Niemelä et al. 2015), and previous work has shown that vibrational stimuli reliably trigger rapid flight into the burrow, validating this response as an ecologically relevant antipredator behaviour (Gilford et al. 2026; Li et al. 2025, 2026).

We conducted field experiments in spring 2025, using *G. campestris* nymphs already present in the meadow from the 24th of April to the 9th of May. In March, we searched the meadow for cricket burrows, which were identified with a uniquely numbered flag. We installed infra‐red HD video cameras over 140 of the burrows, recording activity 24 h a day, allowing behaviours and developmental stages of crickets to be tracked. In mid‐April, crickets began emerging as adults. Upon observing adults with our monitoring system, crickets were caught 2 days post‐emergence into their adult form to allow for pronotum hardening to facilitate tag adhesion, ensuring that all individuals were treated and tested at a consistent early adult stage. When caught, crickets were weighed (±0.01 g), photographed, provided with a treatment (pesticide/sham), and marked with an acrylic tag glued to the pronotum to facilitate individual identification.

### 2.3 Capture and Dosing Protocol

To examine whether sublethal exposure to chlorantraniliprole affects escape behaviours in wild *G. campestris* , newly emerged adults of both sexes were captured in the field and randomly assigned to receive either a topical dose of chlorantraniliprole (35 mg L^−1^; see Appendix 1 for dose‐selection rationale) or a sham acetone treatment. Chlorantraniliprole dilutions were prepared using powdered chlorantraniliprole (PESTANAL analytical standard; CAS No. 500008‐45‐7; Sigma‐Aldrich, Supelco), weighed to the nearest 0.1 mg with an analytical balance (Mettler Toledo AG245; Mettler Toledo, Switzerland), and diluted to 35 mg L^−1^ in 100% acetone. A total of 294 adult crickets were treated: control individuals (*n* = 146) received a topical sham dose of 2 μL acetone, while pesticide‐treated individuals (*n* = 148) received 2 μL of the chlorantraniliprole solution. We applied treatments using a Gilson MICROMAN M10E positive displacement pipette (Gilson, Villiers‐le‐Bel, France). As with the laboratory bioassay, crickets were gently restrained using queen bee marking cages, and individuals were held briefly until the solvent droplet had visibly dried before subsequent tagging and release. For the present study, a subset of 59 individuals was randomly selected for behavioural assays (32 pesticide treated: 16 females, 16 males; 27 controls: 12 females, 15 males). Individuals were selected using a stratified random approach. Crickets were grouped by sex and treatment, and a random subset of individuals were selected from each group each day for behavioural testing the following day. This ensured an approximately balanced representation of sex and treatment groups while maintaining random selection within each category. Using such a subset was necessary due to the logistical constraints of repeatedly locating and testing free‐ranging individuals in the field, particularly during a period of high mobility associated with breeding activity (Rodríguez‐Muñoz et al. 2010), which limited the number of individuals that could be reliably followed and assayed. Post‐dosing, we returned crickets to the same burrows from which they were captured. Previous work in this population has shown that crickets handled and temporarily moved in this way resume normal activity on return to their burrows, with no detectable adverse effects (Rodríguez‐Muñoz et al. 2010, 2019, 2025).

### 2.4 Behavioural Assay and Stimulus Delivery

We used a single‐trial antipredator assay that simulates a predator attack via a standardised vibrational cue, following established protocols (Gilford et al. 2026; Li et al. 2025, 2026). We dropped a 42 mm, 9 g cork ball vertically through a 50 cm tube mounted on a tripod, landing approximately 10 cm in front of the focal cricket burrow. A GoPro camera (2.7 K resolution, 240 fps) was positioned directly above the burrow to record each trial, with a 30 cm ruler placed next to the burrow entrance to provide a spatial scale for distance and speed measurements. Prior to each trial, we measured the body temperature of the focal individual using an infrared thermometer (Testo 830‐T4), as escape performance in ectotherms is known to be temperature‐dependent (Li et al. 2025). We quantified two antipredator behaviours: escape speed and re‐emergence latency. Escape speed (m s^−1^) was quantified from video recordings (see Video Analysis below). Re‐emergence latency (s) was recorded as the interval between a cricket entering its burrow following disturbance and its subsequent full re‐emergence, following the protocol of Gilford et al. (2026). Re‐emergence latency was recorded directly during field observations using a stopwatch rather than extracted from video recordings.

Behavioural testing began 1 day after pesticide or sham dosing, allowing for capture recovery and pesticide absorption. Sublethal effects of insecticides are commonly observed within hours to days following exposure, particularly for compounds that act on neuromuscular systems (Biondi et al. 2013; Desneux et al. 2007). Chlorantraniliprole acts as a ryanodine receptor modulator, disrupting calcium regulation in muscle cells (Dinter et al. 2010), and is therefore expected to affect locomotor performance on relatively short timescales (Cordova et al. 2006; Nauen 2006). Consistent with this, our laboratory bioassay (see bioassay results below) showed that mortality began to emerge within 24 h post‐exposure, indicating that this interval is sufficient for measurable biological effects to occur and therefore represents a plausible window in which behavioural effects could also arise. We therefore used a 1‐day delay as a standardised acute exposure window, while also testing individuals on a second day post‐treatment to capture any short‐term delayed effects. This design was not intended to capture longer‐term or chronic responses, which may emerge over extended periods or under repeated exposure. As adult crickets emerged on different dates, dosing and subsequent test days were staggered across individuals. Consequently, “Day 1” and “Day 2” post‐treatment refer to time since dosing, rather than to the same calendar days. We tested crickets once per day, with a maximum of two consecutive test days per individual if they remained present at a burrow. Of the total sample, 24 individuals were tested on Day 1 only, two on Day 2 only and 33 on both days. Burrow occupation was tracked using infra‐red cameras, positioned over 140 of the burrows.

### 2.5 Video Analysis

Escape speed was quantified from video recordings using ‘Kinovea’ (https://www.kinovea.org/download.html). Video recordings were analysed manually using frame‐by‐frame inspection, with gaps between each frame of 1/240th of a second. We calculated escape speed over the first 1.5 cm of movement, as this provides a standardised estimate of initial response intensity before acceleration introduces variation. All crickets in this study fled at least 1.5 cm, making this a reliable and comparable segment across individuals. This approach follows previous experiments at the same field site, which used the same vibrational stimulus and found 1.5 cm to be sufficient for capturing early escape behaviour while minimising variation due to turning angle or distance to refuge (Li et al. 2025). The following video frames were identified manually; (1) the video frame in which the cricket's first obvious movement was detected, which we denote as the response frame (*r*_*f*_), (2) the video frame in which the stressor‐related cue was released (release frame), (3) the frame in which the cricket reached a distance of 1.5 cm relative to its original position (*t*_*1*_) and (4) the frame that contained the maximum distance fled. Parameters were used to calculate escape speed (m/s). We calculated the time taken for a cricket to cover 1.5 cm (*t*_*x*_) using *t*_*x*_ = (*t*_*1*_ *− r*_*f*_) * (1/240). This was then calculated across the 1.5 cm segment to calculate the escape speed in meters per second (*f*_*s*_); *f*_*s*_ = 0.015 / *t*_*x*_.

### 2.6 Statistical Analyses

We fitted a multivariate Bayesian model to test whether escape speed and re‐emergence latency differed between treatment groups (pesticide vs. sham) and whether these behaviours were affected by days post‐dosing (1 vs. 2 days). The model included two response variables: (1) escape speed and (2) re‐emergence latency (log‐transformed to improve normality). Fixed effects were treatment, days since treatment, sex, and mean‐centred temperature, with individual ID (tag) included as a random intercept to account for repeated measures. Residual correlation between responses was estimated.

To assess whether pesticide effects varied with time since exposure, we compared models with and without a treatment × day interaction using leave‐one‐out cross‐validation (LOO). Models were fitted using the *brms* package (v2.21.0) (Bürkner 2017) under R (v.4.3.2) (R Core Team 2023) using the rstan backend (Stan v2.32.2). Following Bayesian convention, we describe effects as “credible” when their 95% posterior credible intervals do not include zero. We used default brms priors (flat priors on fixed effects and weakly informative priors on intercepts, variance components, and residual correlations) and confirmed via sensitivity checks that inferences were robust to alternative prior specifications (see Appendix 1 Methods). Gaussian likelihoods were used for both traits, following inspection of residual distributions and posterior predictive density‐overlay checks comparing observed and model‐generated distributions, and because alternative distributions did not improve model fit. Parameters were estimated using four MCMC chains (2000 iterations each; 1000 warmup), and convergence was assessed using R‐hat statistics (with values close to 1 indicating good mixing) and effective sample sizes (ESS > 1000). Posterior predictive checks stratified by days post‐treatment verified that the models captured behavioural distributions well. LOO comparisons showed no improvement in predictive performance when the interaction was included (Table A1); therefore, we present results from the additive model.

### 2.7 Ethics

This study was approved by the University of Exeter Research Ethics Panel (approval number: 9802182). A total of 294 individuals were caught and either given a pesticide or sham treatment during the field season. Antipredator behaviour assays reported in this study were conducted on a randomly selected subset of 59 individuals across 92 observations. The use of this subset was necessary due to the logistical challenges of repeatedly locating and testing free‐ranging individuals during the breeding season, when crickets frequently move between burrows in search of mates (Rolando Rodríguez‐Muñoz et al. 2025), limiting the number of individuals that could be reliably followed throughout the behavioural testing period. We carefully selected pesticide treatments based on prior laboratory work to ensure sublethal pesticide exposure levels and minimise mortality. All handling procedures were refined to minimise stress, and individuals were returned to their original burrows immediately following treatment and tagging. We subsequently monitored all crickets in the wild throughout their natural lives. While pesticide exposure may have caused behavioural or physiological changes, no procedures were performed that would be expected to cause pain, injury, or lasting distress.

## 3 Results

**FIGURE A1:**
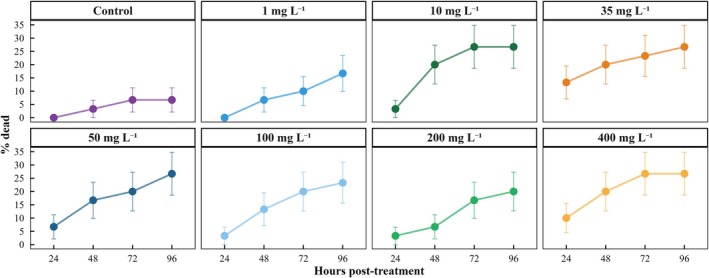
Laboratory bioassay results showing mortality rates of G. bimaculatus following a topical exposure to chlorantraniliprole. Percentage mortality was recorded across 24–96 h post‐treatment for an acetone control and seven pesticide exposure concentrations. Error bars represent standard errors. The concentration selected the field study (35 mg/L^−1^) produced minimal but measurable mortality over the observation period. Three biological replicates were conducted per concentration (*N* = 3), each consisting of 10 individuals (*n* = 10), giving a total of 30 individuals per concentration.

The preparatory laboratory bioassay used to inform dose selection showed that mortality generally increased with chlorantraniliprole concentration and exposure duration, while mortality in acetone controls remained low throughout the observation period (Figure A1). The 35 mg L^−1^ concentration selected for field testing produced low but measurable mortality over 96 h and was therefore retained as a low‐mortality, residue‐level exposure for the field experiment (see Appendix 1).

### 3.1 Do Pesticides Alter Escape Speed and Re‐Emergence Latency?

**FIGURE 1:**
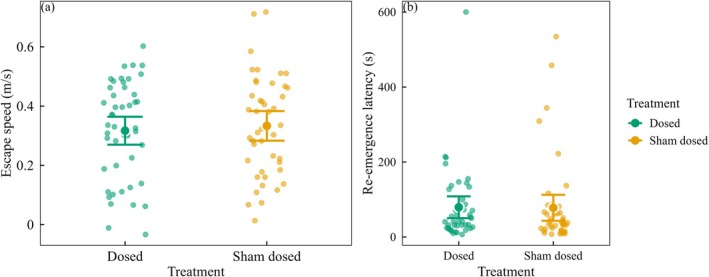
Effects of pesticide exposure on escape speed and re‐emergence latency in adult crickets (*n* = 92). (a) Escape speed (m/s) and (b) re‐emergence latency (s) of crickets either dosed with pesticide (green) (*n* = 47) or sham‐dosed (orange) (*n* = 45). Points represent individuals. Large points with error bars show posterior means and 95% credible intervals.

**FIGURE 2:**
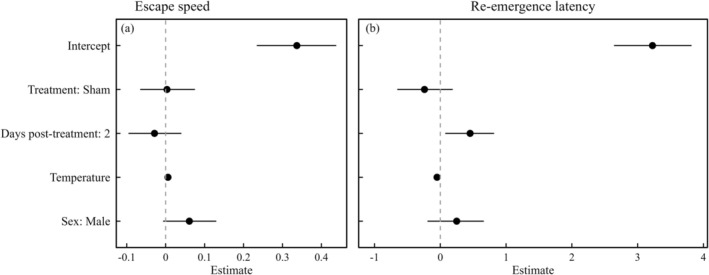
Forest plot of fixed effect estimates for escape speed and re‐emergence latency. Posterior mean estimates (black dots) and 95% credible intervals (horizontal lines) for the fixed effects: Treatment (sham‐dosed), day post‐treatment (Day 2), temperature, and sex (male), shown for (a) escape speed and (b) re‐emergence latency. The vertical dashed line at zero represents no effect.

Crickets exposed to the operationally sublethal pesticide treatment showed no statistically credible difference in escape speed compared to sham‐dosed individuals (*β*_treatment: sham_ = 0.00, 95% CI [−0.07, 0.08]; Figures 1B and 2, Table A2). On the original scale, the estimated mean difference between groups was small (Sham − Dosed: median = 0.003 m s^−1^, 95% CrI [−0.065, 0.075]). In addition, there was no difference in re‐emergence latency between pesticide dosed and sham controlled individuals, as the confidence interval included zero (*β*_treatment:sham_ = −0.24, 95% CI [−0.65, 0.19]; Figures 1A and 2, Table A2); back‐transformed to seconds, the estimated group difference was −8.5 s (Sham − Dosed; 95% CrI [−24.3, 6.9]), which was small relative to natural variation and not credibly different from zero. Posterior mean estimates and 95% credible intervals for all fixed effects are summarised in Figure 2. Model‐predicted behavioural responses across treatments and days post‐treatment are shown in Figure A3 (see Appendix 1).

### 3.2 Does Time Since Treatment Influence Behaviour?

**FIGURE 3:**
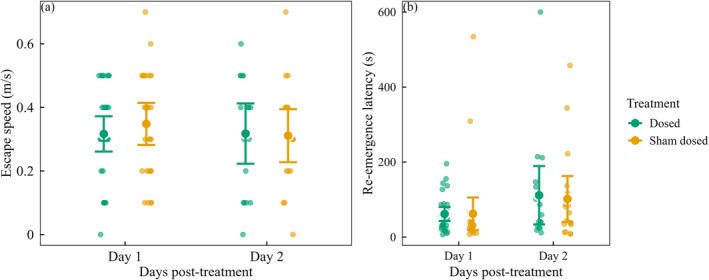
Effects of time since pesticide treatment on escape speed and re‐emergence latency (*n* = 92) Posterior mean (a) escape speed (b) and re‐emergence latency for crickets dosed with pesticide (green) (*n* = 47) or sham dosed (orange) (*n* = 45), measured 1 and/or 2 days after treatment. Points represent individuals. Larger dots and error bars show the posterior mean and associated 95% credible intervals for each group.

**FIGURE A2:**
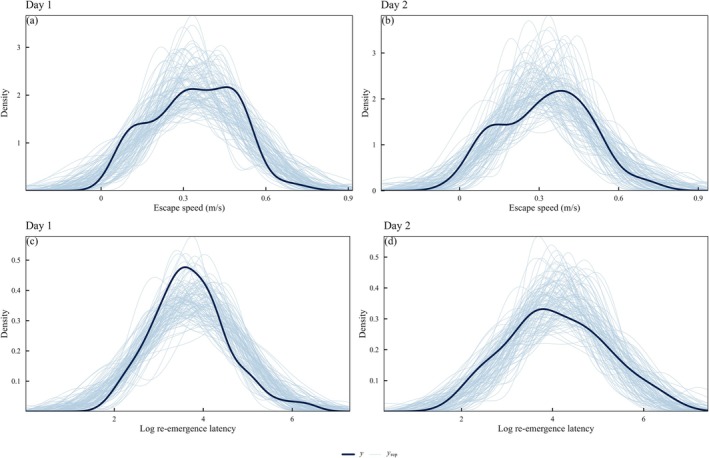
Posterior predictive density plots for escape speed and log‐transformed re‐emergence latency across days post‐treatment in the rsmodel_add model. Plots show observed data distributions (dark lines) and 100 posterior draws (light lines) for: (A) escape speed on Day 1, (B) escape speed on Day 2, (C) log re‐emergence latency on Day 1, and (D) log re‐emergence latency on Day 2. The model captures the central tendencies and spreads of behavioural responses across both days, despite differences in sample size (Day 1 = 57, Day 2 = 35). These plots support the model's ability to reproduce the observed data structure across the two time points.

**FIGURE A3:**
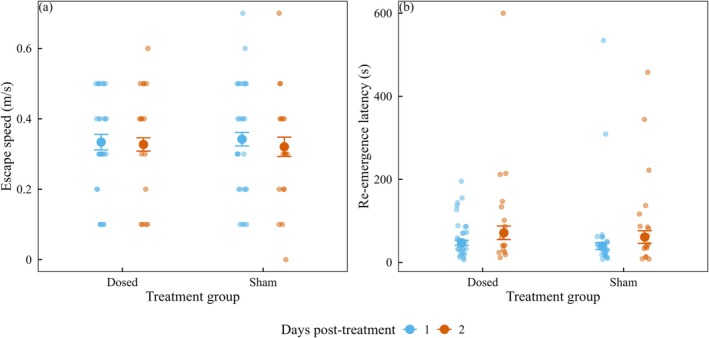
Posterior predictive plots, showing predicted behavioural responses from rsmodel_add presented in Table A1 across 2 days post‐treatment (Day 1 = blue; Day 2 = orange) for crickets dosed with either a sublethal pesticide treatment (Dosed) or a sham control (Sham). Large points represent posterior mean estimates of the fixed effects; vertical lines show 95% credible intervals. Jittered points indicate observed values used in the model. Plots show: (A) escape speed and (B) re‐emergence latency. Predictions were generated with other covariates (temperature and sex) held constant at reference values (mean temperature and female sex) and exclude random effects. All values are shown on the original scale.

Posterior predictive checks stratified by days post‐treatment indicated good model fit (Figure A2), with the models accurately capturing the distributions of both escape speed and log re‐emergence latency for Day 1 (*n* = 57) and Day 2 (*n* = 35), despite the smaller sample size on Day 2. Crickets tested on Day 2 showed longer re‐emergence latencies (*β*_post‐treat:2_ = 0.45, 95% CI [0.08, 0.82], Table A2), corresponding to an increase of approximately 18 s on the original scale (median; 95% CrI [3, 40]), whereas escape speed did not differ by day (*β*_post‐treat:2_ = −0.03, 95% CI [−0.10, 0.04], Table A2) (Figure 3).

### 3.3 Do Other Factors Affect Escape Behaviours?

In addition to the effect of pesticides on escape behaviour, we also explored the effects of temperature and sex. Temperature had a small but credible effect on both escape behaviours. Individuals tested at higher temperatures emerged more quickly (*β*_temp_ = −0.05, 95% CI [−0.08, −0.02]; Table A2) and showed slightly increased escape speeds (*β*_temp_ = 0.01, 95% CI [0.00, 0.01]; Table A2). Sex showed no credible effect on re‐emergence latency (*β*_sex:male_ = 0.25, 95% CI [−0.20, 0.66], Table A2) or escape speed (*β*_sex:male_ = 0.06, 95% CI [−0.01, 0.13]; Table A2).

## 4 Discussion

### 4.1 Do Pesticides Alter Re‐Emergence Latency and Escape Speed?

Our field experiment found no statistically credible effects of sublethal chlorantraniliprole exposure on re‐emergence latency or escape speed in wild field crickets. Under the specific conditions tested here, involving a single, acute, residue‐level exposure measured over a two‐day period, we found no evidence of behavioural impairment in these traits. Our findings are best interpreted as evidence that this particular exposure scenario did not measurably alter the tested components of antipredator behaviour. This does not exclude the possibility of behavioural effects under different exposure regimes, doses or timescales. While the posterior estimates for both escape speed and re‐emergence latency were centred close to zero, the associated uncertainty means that only relatively large behavioural effects could be confidently excluded. Smaller effects, which may still be ecologically relevant, cannot be ruled out.

In addition, although the applied dose was calibrated to be biologically active based on laboratory assays, we cannot directly confirm the extent to which this exposure affected physiological pathways in the focal species under field conditions. If this exposure did not substantially affect neuromuscular or physiological function, then behavioural responses such as escape performance may not be expected to change. This interpretation is consistent with broader work showing that behavioural effects often depend on disruption to underlying physiological systems and may emerge only under sufficiently strong, prolonged or context‐dependent exposure conditions (Adamo 2012; Siviter et al. 2018), although rapid behavioural effects have been reported in some systems, particularly pollinators (Stanley, Smith, et al. 2015). Moreover, experimental studies in other taxa demonstrate that pesticide effects can interact with additional stressors, such as nutritional limitation, to produce behavioural or survival impacts that are not evident under single‐stressor conditions (Ågerstrand et al. 2020; Tosi et al. 2017).

Many laboratory studies report sublethal pesticide‐induced reductions in movement or escape responses across a range of insects, although outcomes vary widely across species, compounds and exposure regimes (Delpuech et al. 2005; Stanley, Smith, et al. 2015; Suchail et al. 2001). However, our study does not directly compare laboratory and field conditions and therefore cannot determine whether differences in outcomes arise from environmental complexity, exposure regime or other factors. The absence of strong acute effects in our study may reflect several non‐exclusive explanations, including intrinsic compensatory capacity in wild crickets, where physiological or behavioural mechanisms help individuals maintain performance after low‐level exposure. It may also reflect environmental buffering, where natural microhabitat conditions such as temperature variation, shelter, or access to resources mitigate the realised toxicity of pesticides compared to simplified laboratory environments (Bro‐Jørgensen et al. 2019; Gressel 2011). Alternatively, sublethal effects may require more prolonged or repeated exposure to manifest (Sanchez‐Bayo and Goka 2014; Siviter et al. 2018). These mechanisms were not directly tested here and remain important avenues for future work.

At the behavioural level, re‐emergence latency increased modestly from Day 1 to Day 2 across both treatment groups, likely reflecting short‐term behavioural plasticity rather than pesticide effects. This interpretation is supported by the absence of a treatment × day interaction, with both sham and pesticide‐exposed individuals showing similar increases in emergence latency. Moreover, previous work at the same field site shows that repeated disturbance reliably leads to elevated re‐emergence latencies in wild crickets (Gilford et al. 2026), consistent with a plastic, experience‐dependent response. This suggests that re‐emergence latency is a flexible, risk‐sensitive trait influenced by recent experience, whereas escape speed appears more constrained and physiologically stable. Minor effects of temperature and sex were consistent with previous work showing that environmental conditions and individual state modulate antipredator behaviour (Li et al. 2025).

### 4.2 Implications and Limitations

Despite modest or null effects, this study provides, to our knowledge, the first field‐based test of sublethal chlorantraniliprole effects on antipredator behaviour in a wild insect population. A key advantage of this approach is that it captures behavioural responses expressed under natural levels of environmental variation, including fluctuations in temperature and predator exposure, which are difficult to replicate simultaneously under laboratory conditions. This allows effect sizes to be estimated in an ecologically relevant context, even when those effects are small or absent, and complements laboratory approaches that identify mechanistic effects under controlled conditions. Against a backdrop of rising diamide use and ongoing insect declines, such field‐based behavioural assays are important for validating laboratory‐derived hazard predictions and understanding how environmental variability shapes behavioural responses in natural systems (Cinel et al. 2020; Sánchez‐Bayo and Wyckhuys 2019).

Several limitations should be considered when interpreting these findings. This study focused on a single, acute, residue‐level exposure measured over a two‐day period, and therefore does not capture the full range of exposure scenarios that insects may experience in natural systems, including repeated or chronic exposure. The exposure regime was designed to represent a residue‐level exposure scenario (~70 ng per cricket), approximately nine‐fold lower than the estimated pesticide load that would be received under direct spray at the recommended field formulation application rate. Consequently, our findings are most applicable to indirect exposure pathways such as spray drift and environmental deposition rather than direct spray events, although exposure levels in natural systems are likely to vary and may occasionally exceed those tested here.

The exposure concentration was selected using a laboratory mortality bioassay in a closely related species (see Appendix 1). While *G. bimaculatus* and *G. campestris* are closely related and capable of producing fertile hybrid offspring (Tyler et al. 2013; Veen et al. 2013), interspecific differences in sensitivity to insecticides are well documented (Arena and Sgolastra 2014; Rondeau et al. 2014), and the relationship between dose and biological effect may therefore differ between species. In the absence of species‐specific toxicity data, we treated the use of *G. bimaculatus* as a working hypothesis for exposure calibration rather than evidence of equivalent pesticide sensitivity between species. The exposure level should therefore be interpreted as a laboratory‐calibrated approximation of a low‐mortality dose in *G. campestris* , rather than a precise estimate of species‐specific sensitivity.

The pesticide‐exposure history of the study population should be considered when interpreting our findings. The focal population forms part of the Wild Crickets Project field site, which has been continuously monitored since 2006, and no pesticides have been applied within the study meadow during this approximately 20‐year period. Land management records further indicate that the meadow has remained free from pesticide application since at least 1972 (*pers. comms*, Rolando Rodríguez‐Muñoz). Direct exposure of the focal population to chlorantraniliprole at the study site is therefore highly unlikely. Although *G. campestris* individuals are capable of dispersal, with movements of up to approximately 284 m recorded over an individual's lifetime (Ritz and Köhler 2007), the species is flightless and populations exhibit substantial genetic structuring and isolation by distance, indicating limited gene flow among populations (Tregenza et al. 2021). Consequently, direct exposure within the focal meadow is unlikely, but prior exposure elsewhere in the surrounding landscape cannot be completely excluded. Populations occupying more agriculturally intensive landscapes may experience different exposure histories, which could influence sensitivity to pesticide treatments or the potential for local adaptation. Future studies comparing populations across gradients of agricultural exposure would help determine the extent to which prior exposure history modifies behavioural responses to chlorantraniliprole.

Both our laboratory bioassay and field experiment used technical‐grade chlorantraniliprole, rather than a commercial formulation. The exposure concentration was derived from the recommended application rate of Coragen. However, commercial pesticide formulations contain additional co‐formulants of undeclared identities and concentrations that can influence uptake, bioavailability and toxicity (Mullin et al. 2016). In some systems, formulated products have been shown to produce stronger biological effects than equivalent concentrations of the isolated active ingredient (Mullin 2015; Straw and Brown 2021). Consequently, the exposure used here is not expected to directly reflect the effects associated with field exposure to formulated chlorantraniliprole products at the same concentration. Our findings should therefore be interpreted as reflecting the effects of the active ingredient itself under a residue‐level exposure scenario, and behavioural effects under exposure to commercial formulations may differ. Nevertheless, isolating the active ingredient provides an important first step in understanding the behavioural consequences of chlorantraniliprole exposure, free from the potentially confounding effects of formulation additives. Future work comparing technical‐grade and commercial formulations under field conditions would help determine the extent to which co‐formulants modify behavioural responses in wild insects.

Our sample size of 59 places a limit on our statistical power to detect subtle effects of pesticide exposure, particularly given the substantial among‐individual and environmental variation observed. Achieving high statistical power in ecological and behavioural studies conducted under realistic field conditions is inherently challenging (Nakagawa et al. 2024), and inference will benefit from replication and synthesis across studies.

### 4.3 Future Directions

It is likely that pesticide effects will be strongest immediately after application, particularly for compounds that act directly on neuromuscular systems such as chlorantraniliprole (Cordova et al. 2006; Nauen 2006; Stanley, Smith, et al. 2015). However, sublethal effects may emerge after longer or repeated exposures, particularly when pesticide exposure occurs alongside additional environmental stressors such as elevated temperatures or repeated disturbance, and in some cases across generations (Batool et al. 2024; Sanchez‐Bayo and Goka 2014; Siviter et al. 2018). Future work on wild populations should prioritise longer‐term and repeated exposure designs where feasible, as well as testing a broader range of exposure levels, including near‐application concentrations. Integrating behavioural assays with physiological and mechanistic approaches, such as examining neuromodulatory pathways including octopamine signalling (S. Adamo 2017), will be important for understanding how exposure translates into altered risk responses and for linking individual behavioural responses to population‐level consequences.

In summary, our findings show that acute, residue‐level exposure to chlorantraniliprole did not measurably alter antipredator escape behaviour in wild field crickets under the conditions tested. These results indicate that such exposure is unlikely to produce large behavioural effects over short timescales, although smaller or delayed effects cannot be ruled out. By assessing behaviour under natural levels of environmental variation, this study provides an ecologically grounded estimate of pesticide effects and supports the integration of field‐based behavioural assays into pesticide risk assessment.

## 5 Conclusion

To our knowledge, this is the first field test of sublethal chlorantraniliprole effects on antipredator behaviour in a wild insect. We found no evidence that a single, acute, residue‐level exposure to chlorantraniliprole alters escape performance in this wild population over a two‐day period, although re‐emergence latency exhibited temporal sensitivity, supporting its potential as an early and responsive indicator of sublethal stress in ecological settings.

In an era of accelerating chemical use and biodiversity decline, linking behavioural plasticity to environmental toxicology remains crucial. These findings are specific to the exposure scenario tested and do not exclude the possibility of effects under higher doses, repeated exposure or longer timescales. By testing predictions largely derived from laboratory studies in a natural population, our results provide evidence that, under the conditions tested, sublethal pesticide exposure did not measurably alter the escape behaviours examined, and further studies are warranted. This work contributes to the growing recognition that behavioural traits, particularly those related to decision‐making and antipredator performance, offer functionally relevant windows into how sublethal stressors may influence organismal fitness and ecological interactions.

## Author Contributions

**Emily Gilford:** conceptualization (equal), data curation (lead), formal analysis (lead), investigation (lead), methodology (lead), project administration (equal), visualization (lead), writing – original draft (lead). **Miranda Johnstone:** investigation (supporting), writing – review and editing (supporting). **Mark Pitt:** resources (supporting), writing – review and editing (supporting). **Rolando Rodríguez‐Muñoz:** methodology (equal), project administration (lead), resources (equal), writing – review and editing (supporting). **Chris Bass:** conceptualization (equal), methodology (supporting), resources (equal), writing – review and editing (supporting). **Tom Tregenza:** conceptualization (lead), methodology (equal), resources (equal), supervision (supporting), writing – review and editing (equal). **Bram Kuijper:** conceptualization (equal), data curation (supporting), funding acquisition (lead), supervision (lead), writing – review and editing (equal).

## Funding

This work was supported by the Natural Environment Research Council (grant number NE/S007504/1 to EG).

## Conflicts of Interest

The authors declare no conflicts of interest.

## Supporting information


**Data S1:** analysis_code_R1.

**Data S2:** mort_data.

**Data S3:** pbd_data.

## Data Availability

Behavioural data are available as Supporting Information. Raw video recordings are available upon request.

## Appendix 1

### Methods

#### Laboratory Bioassay for Dose Selection

To select a realistic sublethal chlorantraniliprole dose for use in the field, we ran a preparatory laboratory bioassay on the closely related Mediterranean field cricket, *Gryllus bimaculatus*, which can produce fertile hybrids with *Gryllus campestris* (Tyler et al. 2013; Veen et al. 2013) and is commercially available as reptile food. The focal species of the field experiment, *G. campestris* , is not readily available year‐round for laboratory experiments. *G. campestris* has an annual life cycle (Rodríguez‐Muñoz et al. 2010), with an obligatory diapause where nymphs overwinter and adults occur primarily in late spring and early summer, limiting opportunities for continuous laboratory culture. Despite more than 20 years of work on this study system throughout the WildCrickets project, we have not been able to establish a viable laboratory population of *G. campestris* . In contrast, *G. bimaculatus* can be readily maintained under laboratory conditions, breeds continuously in captivity, and is widely used as a model species in experimental studies of cricket and physiology. We therefore used *G. bimaculatus* to identify a suitable exposure concentration prior to field testing.

Crickets were kept in a controlled environment at 24°C before and after dosing. We weighed technical grade chlorantraniliprole (PESTANAL analytical standard; CAS No. 500008‐45‐7, Sigma‐Aldrich, Supelco) to 0.01 mg accuracy (A&D HR‐100A analytical balance, A&D Company Ltd., Japan) and dissolved it in 100% acetone to create a series of dose concentrations. Using a Burkhard hand micro applicator (Burkard Manufacturing Co. Ltd., Rickmansworth, UK), we administered a 2 μL drop of either solvent only (sham control), or one of seven chlorantraniliprole concentrations, to the dorsal thorax of 240 adult male crickets (30 individuals per concentration). During application, crickets were gently restrained using a queen bee marking cage to standardise positioning and prevent movement. After dosing, individuals were held briefly until the solvent droplet had visibly dried. Cricket mortality was recorded at 24, 48, 72 and 96 h after dosing. Here, mortality refers to the proportion of individuals found dead at each observation time; no time‐to‐event or survival analysis was conducted. All crickets used in the laboratory bioassay were recently moulted *G. bimaculatus* adults (within 5 days of moulting) and were provided with food and water (cat biscuits) throughout the observation period.

Test concentrations were 0 (acetone control), 1, 10, 35, 50, 100, 200 and 400 mg L^−1^ chlorantraniliprole. The 35 mg L^−1^ dose was calculated from the recommended field application rate of the formulated product Coragen (see product label at https://data.fmc‐agro.co.uk/wp‐content/uploads/coragen_gb_500ml_bkl_k‐40715.15.12.16.pdf (Du Pont (UK) Limited 2016)), and the remaining concentrations were chosen to span a broad range around this value and characterise mortality responses across a range of exposure levels. This bioassay was designed to evaluate mortality responses across a range of concentrations surrounding a field‐realistic residue‐level exposure derived from the recommended Coragen application rate.

While interspecific differences in pesticide sensitivity are well documented (Arena and Sgolastra 2014), this approach assumes, as a working hypothesis, that relative dose‐mortality relationships are broadly comparable between these closely related species, such that a concentration producing low mortality in *G. bimaculatus* provides a reasonable approximation of a low‐mortality exposure in *G. campestris* . While interspecific differences in pesticide sensitivity are well documented (Arena and Sgolastra 2014), ecotoxicological risk assessment frequently relies on extrapolation among related taxa when species‐specific data are unavailable (European Food Safety Authority 2013). The purpose of this bioassay was not to estimate species‐specific toxicity thresholds but to identify a conservative low‐mortality exposure level for subsequent testing in the closely related focal species. We therefore used this approach to identify a suitable exposure concentration for subsequent field testing in *G. campestris* .

#### Laboratory Dose–Response Bioassay Results

In insecticide bioassays conducted in the laboratory, mortality of *G. bimaculatus* generally increased with exposure duration and was higher in chlorantraniliprole‐treated groups than in the acetone‐only control (Figure A1). Mortality in the acetone‐only control treatment remained low throughout the experiment, reaching ~6.7% (two individuals) after 96 h and remaining lower than any chlorantraniliprole treatment. The 35 mg L^−1^ concentration produced low but measurable mortality over the 96‐h observation period and was therefore treated as an operationally sublethal exposure.

#### Dosage Selection Calculations

Based on field‐derived exposure estimates, we selected a working concentration of 35 mg L^−1^ chlorantraniliprole for the field experiment. This solution was applied topically at 2 μL per individual, delivering approximately 70 ng of active ingredient per cricket. This dose was chosen as a low, operationally sublethal exposure consistent with residue‐level contamination likely to occur under field conditions. For comparison, the recommended field application rate of chlorantraniliprole for orchard pest control is approximately 35 g active ingredient (a.i.) per hectare, equivalent to about 0.35μg cm^−2^ under uniform deposition. According to the Coragen MAPP 14930 product label (Du Pont (UK) Limited 2016), the formulation contains 200 g L^−1^ chlorantraniliprole and is recommended at 175 mL ha^−1^ in apples and pears, corresponding to 35 g a.i. ha^−1^. Assuming uniform deposition, this corresponds to approximately 0.35 μg cm^−2^. Based on measurements of dorsal surface area from 24 adult field crickets (mean = 1.86cm^2^), a directly sprayed cricket could receive approximately 651 ng chlorantraniliprole. Our experimental dose delivered approximately 70 ng per individual, around nine‐fold lower than this estimated direct‐spray exposure. We therefore interpret the treatment as a residue‐level exposure scenario rather than a direct overspray event. This comparison is based on the active ingredient alone and does not account for formulation additives present in Coragen that may influence uptake or biological activity under field conditions. Consequently, the exposure experienced by crickets following application of the commercial product may differ from that represented by the technical‐grade chlorantraniliprole used here.

Mortality did not increase monotonically across concentrations, and differences among concentrations were generally modest relative to variation among replicates. Consequently, the bioassay was used as a preliminary dose‐selection exercise rather than a formal toxicity assessment. Thus, the concentration selected for field testing was based primarily on field‐derived exposure estimates rather than the apparent shape of the mortality curve.

The 35 mg L^−1^ dose also produced only minimal mortality over 96 h in our preparatory laboratory bioassay, indicating that it was suitable for testing sublethal behavioural effects in the field. The same topical dosing method and 2 μL application volume used in the laboratory bioassay were then applied to *G. campestris* individuals in the field experiment.

#### Data Analysis

Analysis was conducted in R (v4.3.2) (R Core Team 2023). Data and analysis script are available as Supporting Information. The script performs data import, cleaning, and preprocessing, including converting re‐emergence latencies from mm:ss format to seconds and mean‐centred temperature values to facilitate interpretation of model coefficients. The script also produces figures visualising behavioural patterns and posterior estimates, which are included in the manuscript and appendices. Predictive calibration was additionally assessed using leave‐one‐out probability integral transform (LOO‐PIT) ECDF diagnostics for each response; these showed no strong departures from uniformity, indicating adequate predictive calibration. Default brms priors were used, which was appropriate given the lack of strong prior information for treatment effects in this system. These comprised flat priors on fixed effects and weakly informative Student‐t priors on intercepts, variance components, and residual correlations, providing regularisation without overpowering the likelihood. To assess prior sensitivity, we refit the model using more and less regularising priors; posterior estimates for the treatment and day effects were qualitatively unchanged (posterior means and 95% credible intervals were similar, and conclusions were identical). Posterior distributions showed reasonable behaviour and were not truncated at the bounds of the priors, supporting the adequacy of this approach. For ease of interpretation, we additionally report selected model‐implied differences on the original response scales, calculated from posterior expected values and back‐transformed where necessary.

**TABLE A1:** Model comparison using Leave‐One‐Out cross‐validation (LOO) between the additive model (rsmodel_add) and the interaction model including treatment × days post‐treatment (rsmodel_int).

Model LOO comparison	elpd_diff	se_diff
rsmodel_add	0	0
rsmodel_int	−3.0	0.80

*Note:* The table reports the difference in expected log predictive density (elpd_diff) and its standard error (se_diff), relative to the best‐performing model (set to zero). Differences in predictive performance were small (elpd_diff < 4 and elpd_diff/se_diff < 4) indicating no meaningful improvement in predictive accuracy when the interaction was included. We therefore retained the simpler additive model.

### Results

**TABLE A2:** Posterior summaries of the fixed effects from the Bayesian multivariate mixed‐effects model (rsmodel_add) assessing the effects of chlorantraniliprole exposure on antipredator behaviours in wild field crickets.

Fixed effects	Parameter	Estimate	SE	Lower 95% CI	Upper 95% CI	*R*_hat_
Escape speed	Intercept	0.34	0.05	0.23	0.44	1.00
	Treatment: Sham	0.00	0.04	−0.07	0.08	1.00
	Days post‐treatment: 2	−0.03	0.03	−0.10	0.04	1.00
	Sex: Male	0.06	0.03	−0.01	0.13	1.00
	Temperature	0.01	0.00	0.00	0.01	1.00
Re‐emergence latency (log)	Intercept	3.23	0.30	2.64	3.82	1.00
	Treatment: Sham	−0.24	0.21	−0.65	0.19	1.00
	Days post‐treatment: 2	0.45	0.19	0.08	0.82	1.00
	Sex: Male	0.25	0.22	−0.20	0.66	1.00
	Temperature	−0.05	0.02	−0.08	−0.02	1.00

*Note:* The model included escape speed and re‐emergence latency (log‐transformed) as response variables. Predictors were pesticide treatment (sham/dosed), days post‐treatment (1/2), sex (male/female), and temperature. Posterior mean estimates, standard errors, and 95% credible intervals are provided. *R*_hat_ values indicate model convergence, with values close to 1.00 suggesting satisfactory convergence. Intercepts represent the estimated baseline value when all predictors are at their reference levels: sham treatment, one day post‐treatment, female sex and mean‐centred temperature.
